# Subclinical Atherosclerosis Markers of Carotid Intima-Media Thickness, Carotid Plaques, Carotid Stenosis, and Mortality in Community-Dwelling Adults

**DOI:** 10.3390/ijerph17134745

**Published:** 2020-07-01

**Authors:** Chuan-Wei Yang, Yuh-Cherng Guo, Chia-Ing Li, Chiu-Shong Liu, Chih-Hsueh Lin, Chung-Hsiang Liu, Mu-Cyun Wang, Shing-Yu Yang, Tsai-Chung Li, Cheng-Chieh Lin

**Affiliations:** 1 Department of Medical Research, China Medical University Hospital, Taichung 404, Taiwan; t14636@mail.cmuh.org.tw (C.-W.Y.); t6446@mail.cmuh.org.tw (C.-I.L.); 2 Department of Neurology, China Medical University Hospital, Taichung 404, Taiwan; d24943@mail.cmuh.org.tw (Y.-C.G.); greengen@gmail.com (C.-H.L.); 3 School of Medicine, College of Medicine, China Medical University, Taichung 404, Taiwan; liucs@ms14.hinet.net (C.-S.L.); d5496@mail.cmuh.org.tw (C.-H.L.); d13067@mail.cmuh.org.tw (M.-C.W.); 4 Department of Family Medicine, China Medical University Hospital, Taichung 404, Taiwan; 5 Department of Public Health, College of Public Health, China Medical University, Taichung 404, Taiwan; yz123kimo@yahoo.com.tw; 6 Department of Healthcare Administration, College of Medical and Health Science, Asia University, Taichung 404, Taiwan

**Keywords:** intima–media thickness, plaque, stenosis, mortality

## Abstract

Carotid intima–media thickness (IMT), plaque, and stenosis are widely used as early surrogate markers of subclinical atherosclerosis and strong predictors of future deaths and cardiovascular events. Albuminuria is an indicator of generalized endothelial dysfunction that speeds up atherosclerosis. However, previous studies reporting these associations cannot rule out the confounding effect of albuminuria. We aimed to examine the independent and joint relationships between IMT markers and 10-year mortality in community-dwelling Taiwanese adults. This work was a community-based prospective cohort study consisting of 2956 adults aged at least 30 years recruited in 2007 and followed up through 2019. Cox proportional hazard regression models were used to examine associations of these subclinical atherosclerosis markers with mortality. During an average of 9.41 years of follow up, 242 deaths occurred. The mortality rate was 8.70 per 1000 person-years. Compared with those with carotid IMT less than 1.0 mm, persons with severely increased carotid IMT (≥2.0 mm) had an increased risk for death (hazard ratio (HR): 1.79; 95% confidence interval (CI): 1.07, 3.00). Compared with those without carotid plaque, persons with carotid plaque were more likely to have an increased risk for death (1.65; 1.21–2.32). Compared with those with carotid stenosis less than 25%, persons with carotid stenosis of 25–36% had a significant increased risk for death (1.57; 1.12–2.22). Considering these three IMT markers along with the traditional risk factors (c-statistic: 0.85) significantly increased their predictive ability of mortality compared with any individual variable’s predictive ability (all *p*-values < 0.001 for comparisons of c-statistic values). Carotid IMT measures, including IMT thickness, carotid plaque, and carotid stenosis were significant independent predictors of mortality. Our study supports evidence of blood pressure-related media thickening markers to assess future mortality risks in Chinese adults of general population.

## 1. Introduction

Cardiovascular disease (CVD) is the number one cause of death globally, causing an estimated 17.9 million deaths each year, one-third of which occur prematurely in persons aged 70 years and younger [1]. The American Heart Association suggests carotid artery ultrasound, a noninvasive imaging test, for the evaluation of cerebrovascular and cardiac disease risks. It images arteriosclerosis that characterizes subclinical CVD burden, including the progressive luminal narrowing of the arteries, arterial stiffening, wall thickening, and plaque formation before a person manifests the clinical symptoms of CVD [2].

Carotid intima–media thickness (IMT), a noninvasive, B-mode ultrasound-based measure of the carotid artery by ultrasonography, is a well-established and commonly used early surrogate marker of subclinical atherosclerosis [3]. Carotid IMT predicts CVD incidence or death in the participants of the atherosclerosis risk in communities (ARIC) study recruited from four communities in the United States of America [4] and in asymptomatic persons with at least three CVD risk factors from five European countries [5]. Carotid IMT modestly improves the prediction of CVD death or all-cause mortality after considering the traditional risk factors in participants of the Cardiovascular Health Study (CHS) [6] and in a single-center elderly population community in the Netherlands [7]. Carotid IMT has also been examined in some special populations, such as persons with type 2 diabetes and chronic kidney disease [8], those with peripheral artery disease [9], and HIV-positive persons [10]. 

To the best of our knowledge, no studies have examined the incremental value of IMT along with the inflammatory marker of hs-CRP, and a novel cardiovascular risk factor of urinary albumin–creatinine ratio (ACR) in addition to traditional factors of all-cause mortality in the general population. The aim of the current study was to estimate the independent and joint associations of IMT markers and additional predictive ability of IMT markers along with hs-CRP and ACR in the prediction of all-cause mortality in community-dwelling Taiwanese adults.

## 2. Materials and Methods 

### 2.1. Study Design and Study Subjects

The participants were from the second wave of data in 2007 for Taichung Community Health Study (TCHS) and TCHS for family (TCHS-F), a single-center population-based prospective cohort study including providing extracranial carotid artery ultrasound measurement and a fasting blood sample. A total of 2359 participants (66.83% overall response rate) were selected for TCHS from residents aged 40 years and older in Taichung City, Taiwan in 2004. TCHS-F was a family study. TCHS-F had enlisted 807 families with more than one first-degree relative of TCHS participants. TCHS-F specified the inclusion criteria of adults who were spouses or first-degree blood relatives aged 20 years or older. A total of 1919 family members participated. The third wave of data collection for TCHS and the first wave of data collection for TCHS-F included IMT measurements. In the present prospective cohort study, we specified those who were aged at least 30 years, and 2956 persons were eligible. This study was approved by the Human Research Committee of China Medical University Hospital (CMUH105-REC1-002), all methods were carried out in accordance with relevant guidelines and regulation, and informed consent was obtained from each participant.

### 2.2. Measurements

We used a physical check-up procedure and a standardized questionnaire to complete data collection for sociodemographic factors, lifestyle behaviors, disease histories, and medication history was collected. We performed anthropometric measurements and collected blood samples. Weight and height were measured using an autoanthropometer (superview, HW–666) with the subjects barefoot and wearing light clothing. Body mass index (was derived by weight (kg) over height squared (m^2^). Blood pressure was measured in a seated position using an electronic device (OMRON, HEM-770A, Kyoto, Japan). Blood was drawn in the morning after a 12-h overnight fast with minimal trauma from an antecubital vein. Biochemical markers were analyzed using a biochemical autoanalyzer (Beckman Coluter, Lx-20, Brea, CA, USA), including fasting plasma glucose, triglyceride, HDL-cholesterol, uric acid, urine albumin, and creatinine at the Clinical Laboratory Department of China Medical University Hospital. Urinary ACR was measured using the morning urine sample as a marker of albumin excretion rate. Hs-CRP level was determined by nephelometry, a latex particle-enhanced immunoassay (TBA-200FR, Tokyo, Japan) with inter-assay and intra-assay CVs of <2.0% and 1.9%, respectively; the lower detection limit of the assay was 0.1 mg/L. 

### 2.3. Extracranial Carotid Artery Ultrasound Measurement

IMT measurements were conducted following the standardized procedure by GE Vivid7 color Doppler ultrasound diagnostic apparatus (Vivid 7 Pro, GE, Horten, Norway) by two operators. After taking a rest for approximately 10 min in supine position with the neck in slight hyperextension, all study subjects underwent carotid ultrasound examination with a 10.0 MHz probe to scan the far and near walls of arterial segments (common carotids, bifurcations, and internal carotid arteries). This procedure was performed bilaterally to obtain the transverse and longitudinal (posterior oblique, lateral, and anterior oblique) views. A computer recorded each ultrasound image with an online digital filing system and recorded atherosclerotic plaques and intima–media complex thickness. The maximal IMT of each segment was derived from the mean of three frames of the maximum values of near and far walls on both the left and right sides. Carotid IMT was calculated as a composite measure (maximum of eight carotid sites) that combines the near and far walls of the common carotid arteries IMT, bulb IMT, and internal carotid artery IMT of both sides of the neck. To quantify the degree of stenosis, we defined carotid stenosis as diameter stenosis <25%, 25–36% diameter stenosis, and ≥36% diameter stenosis. The presence or absence of plaque grade was defined as the presence of the intima–media’s focal thickening >1 mm that bulged out into the carotid artery’s lumen with at least twice as thickness as the IMT on either side [11]. The intra-operator reliability of carotid IMT for two operators were 0.85 and 0.97, respectively, and the inter-operator reliability was 0.88.

### 2.4. Statistical Analysis

Simple descriptive analyses of proportion were used for categorical variables and mean with standard deviation for continuous variables. The differences in baseline characteristics between mortality status were evaluated using two-sample *t*-tests for continuous variables and Chi-square or Fisher’s exact tests for categorical variables. We estimated Kaplan–Meier (K–M) mortality cumulative incidence presented as plots, and log-rank test was used to examine the differences in K–M mortality between subgroups of baseline carotid IMT (<1.0, 1.0–2.0 and ≥2.0 mm), carotid plaque (no, yes), and carotid stenosis (<25%, 25–36% and ≥36%). Multivariate Cox proportional hazard models estimated the hazard ratios (HRs) and their 95% confidence intervals (CIs). We tested the assumption of proportionality and the assumption of proportionality was held. The HRs of all-cause mortality stratified by gender were presented, and the interaction terms of IMT markers and gender were examined by likelihood ratio tests with entry of the product terms of each IMT marker and gender into the full models. Restricted cubic splines were applied in Cox models to examine the presence of a dose-response or nonlinear association of IMT markers (carotid IMT and carotid stenosis) as continuous variable with mortality. We explored the joint effect of three IMT markers: carotid plaque, IMT, and carotid stenosis. We estimated the areas under the receiver operating characteristic (AUROC) curves to assess the relative ability of IMT markers in correctly classifying mortality status. The analyses were performed with SAS version 9.4 (SAS, Cary, NC, USA). We considered two-tailed *p*-values, and a *p*-value < 0.05 indicated statistical significance.

## 3. Results

A total of 2956 adults were analyzed. Approximately half of them were male (46.4%). The mean age at baseline of adults who died and were alive were 70.05 years (SD = 11.51 years) and 54.32 years (SD = 11.27 years), respectively. IMT was moderately correlated with carotid plaque and carotid stenosis (point-biserial coefficient *r* = 0.71 and Pearson correlation coefficient *r* = 0.78) and carotid plaque with carotid stenosis (point-biserial correlation coefficient *r* = 0.44). The distributions of baseline characteristics according to mortality status are shown in Table 1. 

**Figure 1:**
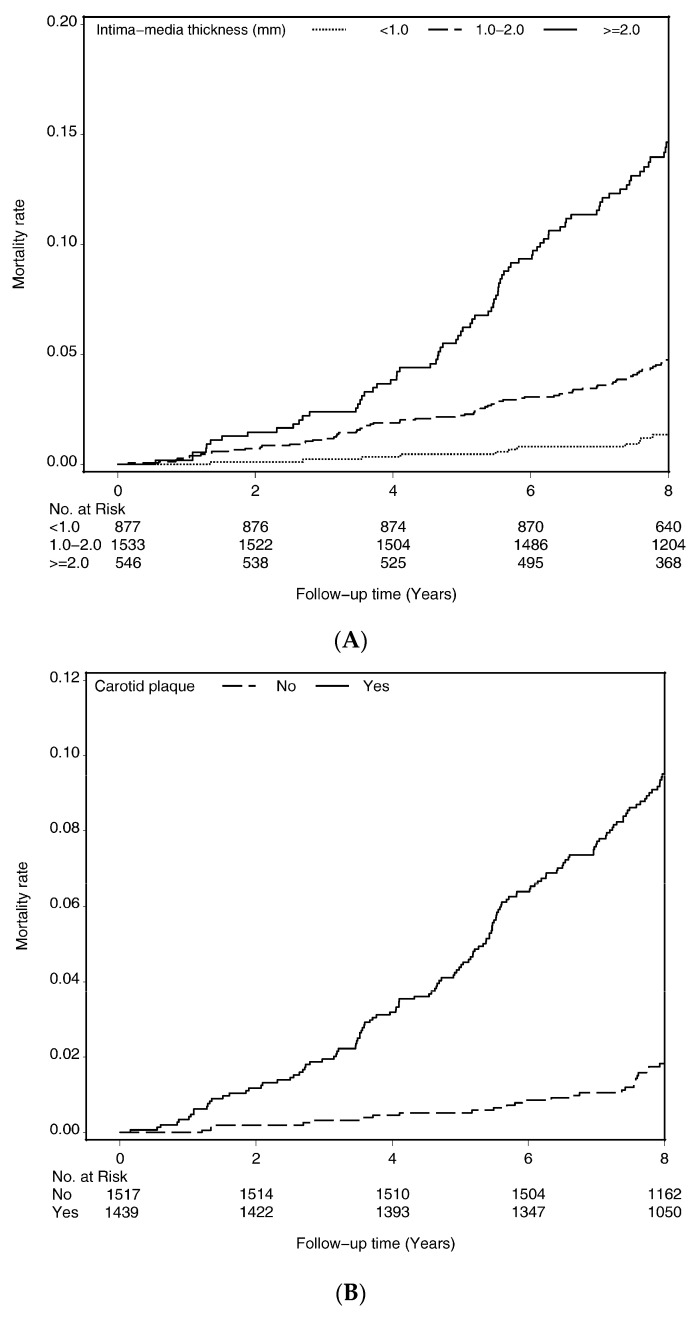

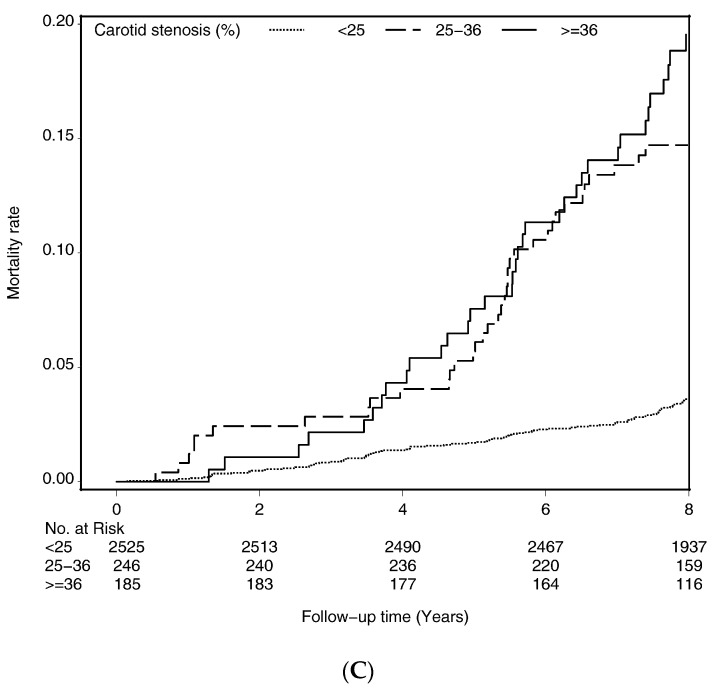
Survival curves of death for (**A**) intima–media thickness, (**B**) carotid plaque, and (**C**) carotid stenosis.

Cumulative incidence curves of death, which was estimated by the Kaplan–Meier method, within subgroups defined by IMT (Figure 1A), carotid plaque (Figure 1B), and carotid stenosis (Figure 1C) were presented. Persons who had higher IMT, carotid plaque, and carotid stenosis had an increased mortality risk (all *p*-values for log-rank tests <0.001). 

**Figure 2:**
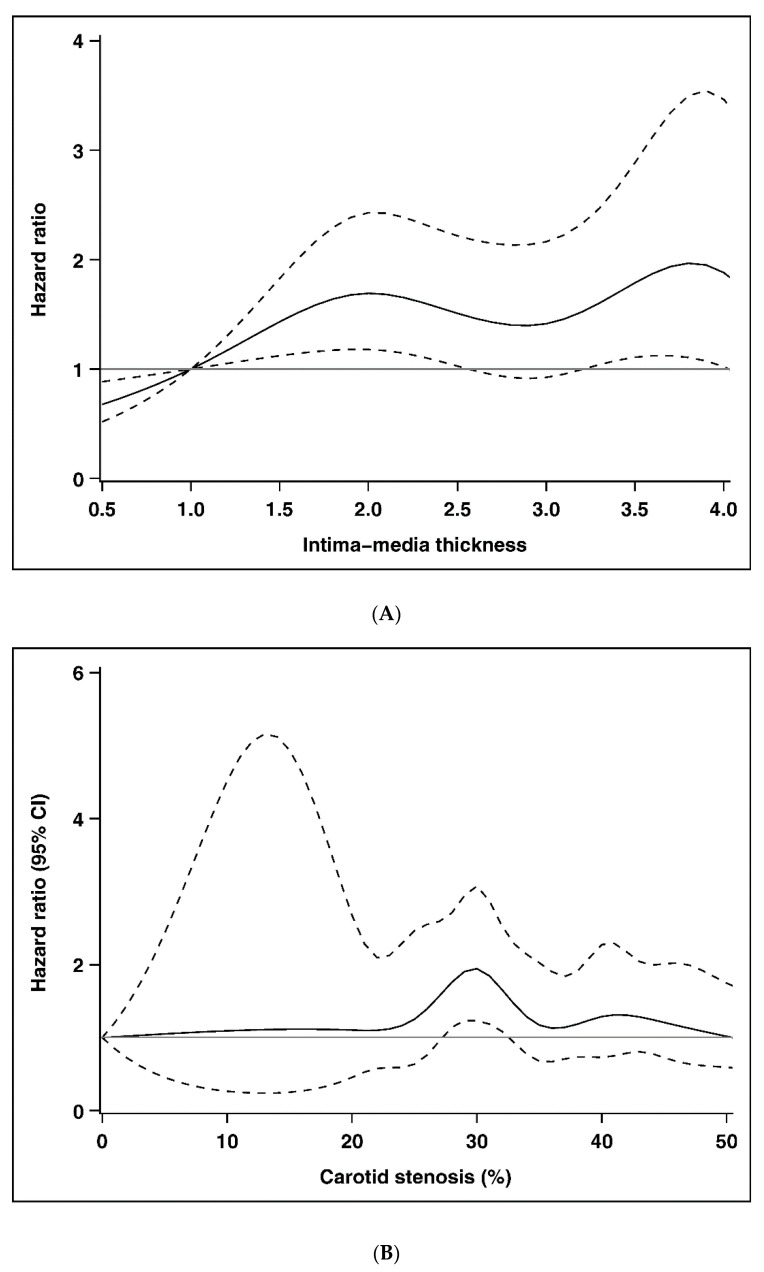
Multivariable restricted cubic spline plots for all-cause mortality by (**A**) intima–media thickness and (**B**) carotid stenosis.

Table 2 presents the incidence density rates and HRs of all-cause mortality according to the subgroups of IMT, carotid plaque, and stenosis. After multivariate adjustment, persons at subgroup of ≥2.0 mm IMT were associated with an increased risk of all–cause mortality (HR [CI]: 1.79 [1.07–3.00]) compared with those with IMT < 1.0 mm. Compared with adults without carotid plaque, the multivariate-adjusted HR (CI) of all-cause mortality was 1.65 (1.17–2.32) for those who had carotid plaque. The mortality risk for persons whose carotid stenosis was 25–36% was 1.57 times higher than those whose carotid stenosis was <25%. Multivariable restricted cubic spline plots for all-cause mortality by carotid IMT and carotid stenosis are shown in Figure 2. The multivariable splines for carotid IMT and carotid stenosis demonstrated nonlinear associations with all-cause mortality. The curve of HRs for IMT appears roughly M shape with two peaks at 2.0 and 3.8 mm and the HR was greatest at 3.8 mm. The HRs for carotid stenosis were close to 1 before 25% and then gradually increased and reached the peak at 30% and then gradually decreased; and there was a turning point at 36% and reached the peak at 43%.

**Figure 3:**
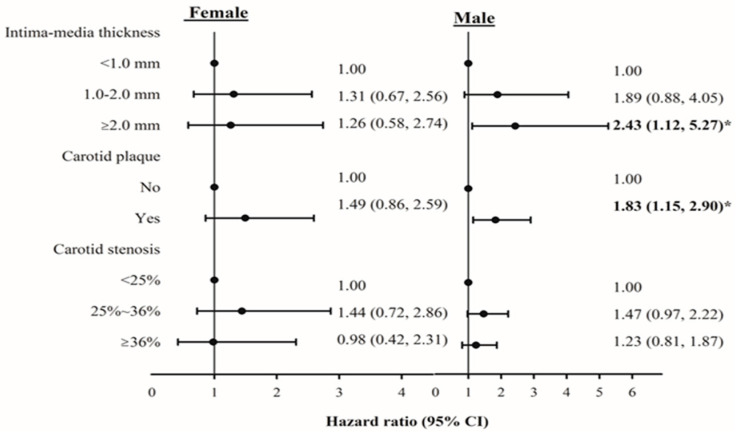
Adjusted hazard ratios (HRs) of mortality stratified by gender. * *p* < 0.05.

**Figure 4:**
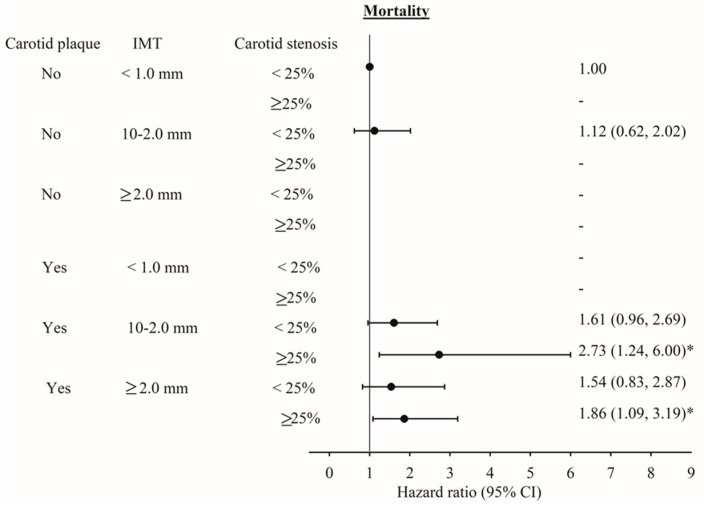
The adjusted HR of mortality the effects of intima–media thickness, carotid plaque, and carotid stenosis. * *p* < 0.05.

Associations among IMT, carotid plaque, stenosis, and all-cause mortality were stratified by gender (Figure 3). The HR of mortality for IMT and carotid plaque in men were slightly higher than those in women. In men, HRs (95% CI) for IMT ≥ 2.0 mm were 2.43 (1.12–5.27, *p* for trend 0.01) and 1.83 (1.15–2.90) for carotid plaque. Given the small sample size, interactions between gender and IMT and carotid plaque were not significant. Figure 4 shows the adjusted HRs of all-cause mortality for the joint effects of carotid plaque, IMT, and carotid stenosis. Owing to the limited number of persons with no plaque, IMT < 2.0 mm but stenosis ≥25% or with plaque, IMT < 1.0 mm, and stenosis <25% or ≥25%, we could not estimate their joint effects. We observed significant HRs of all-cause mortality for persons with plaque, IMT 1.0–2.0 mm or ≥2.0 mm, and stenosis ≥25% compared with persons with no plaque, IMT < 1.0 mm, and carotid stenosis < 25% (HR = 2.73, 95% CI = 1.24–6.00; and HR = 1.86, 95% CI = 1.09–3.19, respectively).

**Figure 5:**
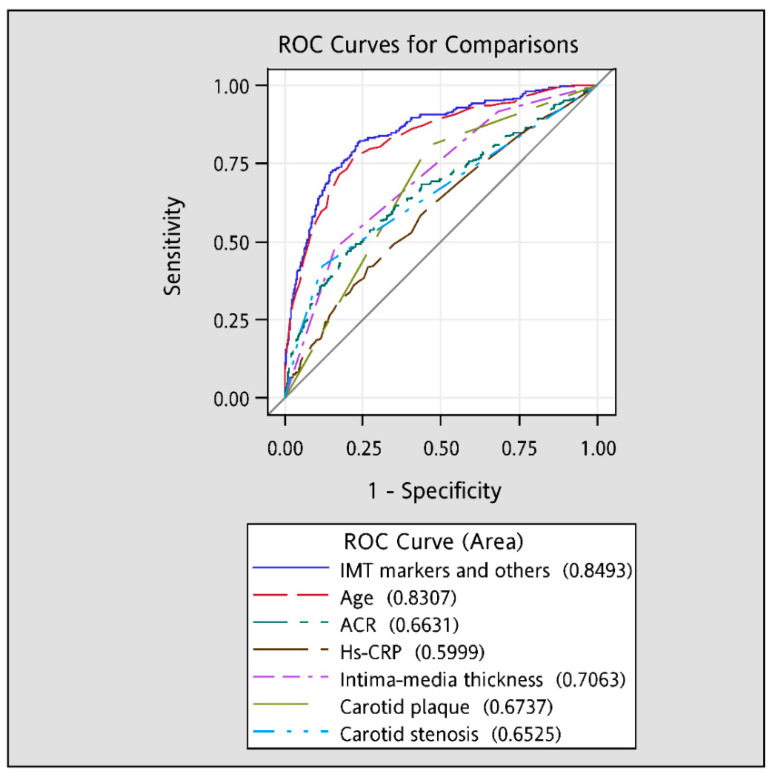
The areas under the receiver operating characteristic (AUROC) curves for all-cause mortality.

The AUROC curves (Figure 5) demonstrated that individual IMT (c-statistic = 0.71) was the only IMT marker with good predictive ability (c-statistic > 0.70), followed by carotid plaque (0.67) and carotid stenosis (0.65). Among all traditional factors, age had the best predictive ability (c-statistic = 0.83), followed by ACR (0.66) and hs-CRP (0.60). Considering these three IMT markers along with the traditional risk factors (0.85) significantly increased their predictive ability of all-cause mortality compared with any individual variable’s predictive ability (all *p*-values < 0.001).

## 4. Discussion

In the present prospective cohort study, we demonstrated that carotid IMT, carotid plaque, and carotid stenosis were all moderately correlated with one another. However, each remained an independent risk factor for all-cause mortality after considering traditional risk factors. In addition, we reported the joint effects of these three IMT markers and found that the magnitude of the strength of joint association was substantially greater than that of individual IMT markers, which will improve the risk stratification for mortality. These three IMT markers along with traditional risk factors resulted in remarkably good ability to predict CVD as measured by the c-statistic.

The quantification of IMT and carotid plaques as measured by ultrasonography is a strong predictor of cardiovascular endpoints in various populations [4,5,6,7,8,10,12]. These prior studies focused on whether additional prediction ability of IMT and carotid plaques on cardiovascular endpoints may serve as an add-on to traditional risk factors, especially hs-CRP. Albuminuria, an indicator of generalized endothelial dysfunction that speeds up atherosclerosis [13], is a predictor marker to evaluate the increased risks of CVD [14,15] or mortality [16,17]. In addition, ACR, a biomarker for measuring albuminuria, is associated with ACR and carotid IMT [18,19]. However, previous studies did not consider albuminuria as a covariate on the association between IMT or plaque measurements and cardiovascular outcomes to rule out its confounding effect. Our data indicated that the AUROC value for ACR was significantly higher than that for hs-CRP. Simultaneously considering these three IMT markers along with the traditional risk factors (c-statistic: 0.85) significantly increased their predictive ability of all-cause mortality.

Two studies conducted among persons of general population from the CHS and ARIC studies found significant associations between IMT measurements and CVD incidence and all-cause mortality [6] or coronary heart disease [4]. Cao identified an association using carotid IMT measurement, which is a composite measure that merges the maximum internal and common carotid wall thickness of the right and left carotid arteries, and explored the independent effects of carotid IMT and plaque [6]. Nambi evaluated whether the Framingham CHD risk score had a significant improvement by using IMT measurement, estimated by the mean of the mean measurements across the segments of carotid artery bifurcation, distal common carotid, and proximal internal carotid arteries for both right and the left sides, and/or the presence or absence plaque over the model with Framingham traditional risk factors [4]. The values of c-statistics, which indicates the discriminatory ability of the models, for all-cause mortality and composite CHD outcomes in the previous studies were 0.7582 and 0.7086, respectively, with consideration of combined carotid IMT and plaque as carotid atherosclerosis along with traditional risk factors. However, in the latter study, the CHD incidence was 0.755. The value of c-statistics in our study was 0.85, which was considerably higher than those in these two studies. Variations in IMT measurements and covariates considered may partially explain why these findings differ from ours. A prior study has shown that vegetable diversity was associated with IMT and 15-year atherosclerotic vascular disease mortality [20]. Different geographical areas with different dietary behaviors may explain the differences. Our study adopted the maximum values of the maximum measurements of the right and left sides across the distal common carotid, carotid artery bifurcation, and proximal internal segments of carotid arteries. In addition to plaque and IMT measurements, we measured carotid stenosis. Although only IMT marker alone had good predictive ability, these three markers were independently associated with all-cause mortality after considering traditional risk factors. Furthermore, the strength of association for the joint variables of these markers was considerably greater than that for individual markers, indicating these three IMT markers improved risk stratification for mortality.

The non-invasive measurement for extracranial carotid artery and internal carotid artery (ICA, carotid bulb) IMT, and plaques to predict cardiovascular risk is an easy and accessible method for the general population [21]. The relative risk of stroke is higher in the patients with extracranial internal carotid artery stenosis than the patients with intracranial artery stenosis [22]. The incidence of stroke by occlusion and artery-to-artery embolism of extracranial internal carotid artery high-grade atherosclerosis is high. The association between intracranial atherosclerotic disease and extracranial atherosclerotic disease has been evaluated in white and Asian patients with previous cardiac diseases or stroke [23]. Stenosis in extracranial common carotid artery and internal carotid artery was associated with more than two times the odds of having stenosis in the intracranial arteries [23]. Thus, IMT measurement has been proposed as a measure of subclinical CVD for assessing global cardiovascular risk by two consensus groups [24,25].

In the present study we provided evidence that increased IMT thickness, carotid plaque, and carotid stenosis were significant independent predictors of mortality in a Taiwanese general population. It has been reported cardiovascular risk factors of systolic blood pressure, high-density lipoprotein, diabetes, and smoking are associated with the mean common carotid artery IMT [26]. Lifestyle changes and therapeutic approaches to carotid atherosclerosis underline the potential clinical implications of our study’s findings. Many lifestyle changes could slow the progression of carotid artery stenosis such as cessation of smoking and tobacco products use, daily physical exercise, body weight control, reduction in dietary calories intake, and low intake in saturated fats, cholesterol, and sodium [27]. Therapeutic approaches such as antihypertensive and antihyperlipidemia drugs have been demonstrated to reduce the atherosclerosis development measured by CIMT [28].

The TCHS study has several strengths. It examined a large number of traditional factors, including hs-CRP and ACR, thereby allowing us to identify the additional predictive ability of IMT for predictive purposes. TCHS was conducted in a community with probability sampling approach. We had standardized approaches for carotid image acquisition. Carotid IMT measurement across operators and all operators were trained and certified. We analyzed all scans blindly in the same reading center. The values for within-operator and between operators were in good agreement. By linking with national death registry resulting in all participants with complete follow-up, “loss to follow-up” bias may have minimally affected our study results. Finally, we considered many potential confounders, such as the inflammatory markers of hs-CRP, WBC, and ACR, in addition to traditional cardiovascular biomarkers in our multivariate analyses.

Our study has some potential limitations. The findings can only be generalized to the general Chinese population. Especially, the HRs we observed were slightly larger than those reported in other Western populations [6] but smaller than in patients with type 2 diabetes and chronic kidney disease [8]. Finally, the low number of cardiovascular events have limited us to explore the effects of IMT on CVD-specific mortality.

## 5. Conclusions

Carotid IMT, carotid plaque, and carotid stenosis each independently contributed to all-cause mortality. Our study supports evidence of blood pressure-related media thickening markers to assess future mortality risks in Chinese adults of general population. The addition of carotid atherosclerosis measures to conventional risk factors resulted in a modest increase in the ability to predict all-cause mortality on the basis of ROC analysis.

## Figures and Tables

**Table 1:** The distributions of baseline socio-demographic factors, lifestyle behaviors, disease history, blood biochemical indexes according to mortality status.

Variables	Mortality Status n (%)		*p*-Value
	Alive (N = 2714)	Dead (N = 242)	
Socio-demographic factors			
Men	1205 (44.4)	167 (69.01)	<0.001
Age (years)	54.32 ± 11.27	70.05 ± 11.51	<0.001
Educational attainment			<0.001
≤6 years	213 (17.15)	57 (31.32)	
7–12 years	527 (42.43)	65 (35.71)	
≥13 years	502 (40.42)	60 (32.97)	
Married	2162 (79.66)	203 (83.88)	0.14
Body mass index (kg/m^2^)			0.61
<18.5	73 (2.69)	9 (3.72)	
18.5–25	1636 (60.28)	141 (58.26)	
25–30	843 (31.06)	74 (30.58)	
≥30	162 (5.97)	18 (7.44)	
Lifestyle behaviors			
Smoking	362 (13.34)	43 (17.77)	0.07
Alcohol drinking	483 (17.80)	56 (23.14)	0.05
Physical activity	1698 (62.56)	167 (69.01)	0.05
*Disease history*			
Hypertension	662 (24.39)	130 (53.72)	<0.001
Diabetes Mellitus	245 (9.03)	63 (26.03)	<0.001
Heart disease	277 (10.21)	71 (29.34)	<0.001
Stroke	37 (1.36)	25 (10.33)	<0.001
Cancer	82 (3.02)	25 (10.33)	<0.001
CKD (eGFR < 60 mL/min/1.73 m^2^)	117 (4.31)	67 (27.69)	<0.001
Blood biochemical indexes †			
Fasting plasma glucose (mg/dL)	100.85 ± 22.34	111.57 ± 33.99	<0.001
Total cholesterol (mg/dL)	199.08 ± 35.28	190.18 ± 36	<0.001
Triglyceride (mg/dL)	121.87 ± 89.75	137.55 ± 95.37	0.01
High-density lipoprotein (mg/dL)	48.88 ± 14.08	44.47 ± 13.09	<0.001
Low-density lipoprotein (mg/dL)	120.94 ± 31.27	112.64 ± 31.81	<0.001
WBC (10^3^/mL)	5.75 ± 1.56	6.12 ± 1.70	<0.001
ACR (mg/g)	21.82 ± 103.34	127.66 ± 532.54	0.002
Fasting insulin (IU/L)	6.74 ± 4.79	7.73 ± 8.47	0.08
Hs-CRP (mg/L)	0.16 ± 0.28	0.27 ± 0.44	<0.001
Measurements of the carotid arteries			
Total carotid intima–media thickness			<0.001
<1.0 mm	857 (31.58)	20 (8.26)	
1.0–2.0 mm	1426 (52.54)	107 (44.21)	
≥2.0 mm	431 (15.88)	115 (47.52)	
Carotid plaque			<0.001
No	1470 (54.16)	47 (19.42)	
Yes	1244 (45.84)	195 (80.58)	
Carotid stenosis			<0.001
<25%	2385 (87.88)	140 (57.85)	
25%–36%	195 (7.18)	51 (21.07)	
≥36%	134 (4.94)	51 (21.07)	

CKD: chronic kidney disease; †: mean (SD). Student’s *t*-test was used for continuous variables to calculate *p*-values. Chi-square test was used for categorical variables to calculate *p*-values.

**Table 2:** Hazard ratios of all-cause mortality according to intima–media thickness, carotid plaque, and carotid stenosis.

Variables	n	Cases	Person-Years	Incidence Rate	Age and Sex-Adjusted	Multivariate-Adjusted ^1^	Multivariate-Adjusted ^2^
					HR (95%CI)	HR (95%CI)	HR (95%CI)
Intima–media thickness							
<1.0 mm	877	20	8140.94	2.46	1.00	1.00	1.00
1.0–2.0 mm	1533	107	14,714.21	7.27	1.58 (0.97, 2.57)	1.53 (0.94, 2.49)	1.53 (0.94, 2.50)
≥2.0 mm	546	115	4955.72	23.21	2.22 (1.34, 3.68) **	2.06 (1.24, 3.42) **	1.79 (1.07, 3.00) *
*p* for trend					<0.001	0.002	0.003
Carotid plaque							
No	1517	47	14,453.17	3.25	1.00	1.00	1.00
Yes	1439	195	13,357.71	14.60	1.89 (1.35, 2.64) ***	1.82 (1.30, 2.55) ***	1.65 (1.17, 2.32) **
Carotid stenosis							
<25%	2525	140	24,074.52	5.82	1.00	1.00	1.00
25%–36%	246	51	2118.61	24.07	1.75 (1.25, 2.46) **	1.71 (1.22, 2.39) **	1.57 (1.12, 2.22) **
≥36%	185	51	1617.74	31.53	1.54 (1.09, 2.18) *	1.49 (1.05, 2.10) *	1.15 (0.80, 1.67)
*p* for trend					0.004	0.007	0.22

* *p* < 0.05; ** *p* < 0.01; *** *p* < 0.001. ^1^ Multivariate adjustment for age, sex, education, marital status, BMI, smoking, alcohol drinking, and physical activity. ^2^ Multivariate adjustment for age, sex, education, marital status, BMI, smoking, alcohol drinking, physical activity, hypertension, diabetes mellitus, heart disease, stroke, cancer, chronic kidney disease, fasting plasma glucose, total cholesterol, triglyceride, high-density lipoprotein, low-density lipoprotein, WBC, ACR, fasting insulin, and hs-CRP. Incidence rate = number of incident cases/person-years * 1000; HR: hazard ratio; CI: confidence interval.

## References

[1] WHO Cardiovascular Diseases. https://www.who.int/health–topics/cardiovascular–diseases/#tab=tab_1.

[2] Blankenhorn D.H., Hodis H.N. (1994). George lyman duff memorial lecture. Arterial imaging and atherosclerosis reversal. Arter. Thromb..

[3] O’Leary D.H., Polak J.F. (2002). Intima–media thickness: A tool for atherosclerosis imaging and event prediction. Am. J. Cardiol..

[4] Nambi V., Chambless L., Folsom A.R., He M., Hu Y., Mosley T., Volcik K., Boerwinkle E., Ballantyne C.M. (2010). Carotid intima–media thickness and presence or absence of plaque improves prediction of coronary heart disease risk: The ARIC (Atherosclerosis Risk In Communities) study. J. Am. Coll. Cardiol..

[5] Baldassarre D., Hamsten A., Veglia F., de Faire U., Humphries S.E., Smit A.J., Giral P., Kurl S., Rauramaa R., Mannarino E. (2012). Measurements of carotid intima–media thickness and of interadventitia common carotid diameter improve prediction of cardiovascular events: Results of the IMPROVE (Carotid Intima Media Thickness [IMT] and IMT–Progression as Predictors of Vascular Events in a High Risk European Population) study. J. Am. Coll. Cardiol..

[6] Cao J.J., Arnold A.M., Manolio T.A., Polak J.F., Psaty B.M., Hirsch C.H., Kuller L.H., Cushman M. (2007). Association of carotid artery intima–media thickness, plaques, and C–reactive protein with future cardiovascular disease and all–cause mortality: The Cardiovascular Health Study. Circulation.

[7] Stork S., Feelders R.A., van den Beld A.W., Steyerberg E.W., Savelkoul H.F., Lamberts S.W., Grobbee D.E., Bots M.L. (2006). Prediction of mortality risk in the elderly. Am. J. Med..

[8] Roumeliotis A., Roumeliotis S., Panagoutsos S., Theodoridis M., Argyriou C., Tavridou A., Georgiadis G.S. (2019). Carotid intima–media thickness is an independent predictor of all–cause mortality and cardiovascular morbidity in patients with diabetes mellitus type 2 and chronic kidney disease. Ren. Fail..

[9] Clemens R.K., Annema W., Baumann F., Roth-Zetzsche S., Seifert B., von Eckardstein A., Amann-Vesti B.R. (2019). Cardiac biomarkers but not measures of vascular atherosclerosis predict mortality in patients with peripheral artery disease. Clin. Chim. Acta; Int. J. Clin. Chem..

[10] Hanna D.B., Moon J.Y., Haberlen S.A., French A.L., Palella F.J., Gange S.J., Witt M.D., Kassaye S., Lazar J.M., Tien P.C. (2018). Carotid artery atherosclerosis is associated with mortality in HIV–positive women and men. Aids (Lond. Engl.).

[11] Chang C.-S., Kuo C.-L., Huang C.-S., Cheng Y.-S., Lin S.-S., Liu C.-S. (2019). Association of cyclophilin A level and pulse pressure in predicting recurrence of cerebral infarction. Kaohsiung J. Med. Sci..

[12] Zhang Y., Fang X., Hua Y., Tang Z., Guan S., Wu X., Liu H., Liu B., Wang C., Zhang Z. (2018). Carotid Artery Plaques, Carotid Intima–Media Thickness, and Risk of Cardiovascular Events and All–Cause Death in Older Adults: A 5–Year Prospective, Community–Based Study. Angiology.

[13] Brevetti G., Schiano V., Chiariello M. (2008). Endothelial dysfunction: A key to the pathophysiology and natural history of peripheral arterial disease?. Atherosclerosis.

[14] Hillege H.L., Janssen W.M., Bak A.A., Diercks G.F., Grobbee D.E., Crijns H.J., Van Gilst W.H., De Zeeuw D., De Jong P.E. (2001). Microalbuminuria is common, also in a nondiabetic, nonhypertensive population, and an independent indicator of cardiovascular risk factors and cardiovascular morbidity. J. Intern. Med..

[15] Weiner D.E., Tighiouart H., Amin M.G., Stark P.C., MacLeod B., Griffith J.L., Salem D.N., Levey A.S., Sarnak M.J. (2004). Chronic kidney disease as a risk factor for cardiovascular disease and all–cause mortality: A pooled analysis of community–based studies. J. Am. Soc. Nephrol. Jasn.

[16] Schmieder R.E., Mann J.F., Schumacher H., Gao P., Mancia G., Weber M.A., McQueen M., Koon T., Yusuf S. (2011). Changes in albuminuria predict mortality and morbidity in patients with vascular disease. J. Am. Soc. Nephrol. Jasn.

[17] Warnock D.G., Muntner P., McCullough P.A., Zhang X., McClure L.A., Zakai N., Cushman M., Newsome B.B., Kewalramani R., Steffes M.W. (2010). Kidney function, albuminuria, and all–cause mortality in the REGARDS (Reasons for Geographic and Racial Differences in Stroke) study. Am. J. Kidney Dis. Off. J. Natl. Kidney Found..

[18] Huang Y., Chen Y., Xu M., Gu W., Bi Y., Li X., Ning G. (2010). Low–grade albuminuria is associated with carotid intima–media thickness in Chinese type 2 diabetic patients. J. Clin. Endocrinol. Metab..

[19] Li M.F., Tu Y.F., Li L.X., Lu J.X., Dong X.H., Yu L.B., Zhang R., Bao Y.Q., Jia W.P., Hu R.M. (2013). Low–grade albuminuria is associated with early but not late carotid atherosclerotic lesions in community–based patients with type 2 diabetes. Cardiovasc. Diabetol..

[20] Blekkenhorst L.C., Lewis J.R., Bondonno C.P., Sim M., Devine A., Zhu K., Lim W.H., Woodman R.J., Beilin L.J., Thompson P.L. (2019). Vegetable diversity in relation with subclinical atherosclerosis and 15–year atherosclerotic vascular disease deaths in older adult women. Eur. J. Nutr..

[21] Naqvi T.Z., Lee M.S. (2014). Carotid intima–media thickness and plaque in cardiovascular risk assessment. JACC Cardiovasc Imaging.

[22] Kappelle L.J., Eliasziw M., Fox A.J., Sharpe B.L., Barnett H.J. (1999). Importance of intracranial atherosclerotic disease in patients with symptomatic stenosis of the internal carotid artery. The North American Symptomatic Carotid Endarterectomy Trail. Stroke.

[23] Suemotoa C.K., Grinbergb L.T., Leitea R.E.P., Ferretti-Rebustinic R.E.L., Jacob-Filhoa W., Yaffed K., Nitrinie R., Pasqualucci C.A. (2018). Morphometric measurements of extracranial and intracranial atherosclerotic disease: A population–based autopsy study. Atherosclerosis.

[24] Stein J.H., Korcarz C.E., Hurst R.T., Lonn E., Kendall C.B., Mohler E.R., Najjar S.S., Rembold C.M., Post W.S. (2008). American Society of Echocardiography Carotid Intima–Media Thickness Task Force. Use of carotid ultrasound to identify subclinical vascular disease and evaluate cardiovascular disease risk: A consensus statement from the American Society of Echocardiography Carotid Intima–Media Thickness Task Force. Endorsed by the Society for Vascular Medicine. J. Am. Soc. Echocardiogr.

[25] Touboul P.J., Hennerici M.G., Meairs S., Amarenco P., Bornstein N., Csiba L., Desvarieux M., Ebrahim S., Fatar M., Hernandez R.H. (2007). Mannheim carotid intima–media thickness consensus (2004–2006): An update on behalf of the Advisory Board of the 3rd and 4th Watching the Risk Symposium, 13th and 15th European Stroke Conferences, Mannheim, Germany, 2004, and Brussels, Belgium, 2006. Cereb. Dis..

[26] Polak J.F., Pencina M.J., Meisner A., Pencina K.M., Brown L.S., Wolf P.A., D’Agostino R.B. (2010). Associations of carotid artery intima–media thickness (IMT) with risk factors and prevalent cardiovascular disease: Comparison of mean common carotid artery IMT with maximum internal carotid artery IMT. J. Ultrasound Med..

[27] Prasad K. (2015). Pathophysiology and medical treatment of carotid artery stenosis. Int. J. Angiol..

[28] Wiklund O., Hulthe J., Wikstrand J., Schmidt C., Olofsson S.O., Bondjers G. (2002). Effect of controlled release/extended release metoprolol on carotid intima–media thickness in patients with hypercholesterolemia: A 3–year randomized study. Stroke.

